# Serial native T1- and T2-mapping to quantitatively monitor resorption of myocardial edema following acute myocardial infarction

**DOI:** 10.1186/1532-429X-17-S1-P101

**Published:** 2015-02-03

**Authors:** Enver Tahir, Martin R Sinn, Ulf K Radunski, Dennis Säring, Christian Stehning, Kai Muellerleile, Bernhard Schnackenburg, Gerhard Adam, Gunnar Lund

**Affiliations:** Diagnostic and Interventional Radiology, University Medical Center Hamburg-Eppendorf, Hamburg, Germany; Institute for Computational Neuroscience, University Medical Center Hamburg-Eppendorf, Hamburg, Germany; General and Interventional Cardiology, University Medical Center Hamburg-Eppendorf, Hamburg, Germany; Philips Research Hamburg, Hamburg, Germany

## Background

Currently, myocardial edema monitoring after acute myocardial infarction (AMI) is based on visualization of the region with increased signal-intensity on T2-weighted images. Native T1 and T2 mapping are promising novel MRI techniques to quantitatively assess myocardial edema. The purpose of the study was to quantitatively evaluate resorption of myocardial edema following AMI by native T1- and T2-mapping cardiac magnetic resonance imaging (CMR).

## Methods

CMR (1.5 Tesla Philips Achieva) was performed in 11 patients four times after reperfused AMI at baseline (BL) at 10 ±7 days after infarction and at 7.2 ±1.4 weeks (follow-up 1, FU1), 3.4 ±0.3 months (FU2) and 6.5 ±0.5 months (FU3), respectively. Edema-sensitive black-blood T2-weighted (T2w) STIR CMR was performed on end-diastolic LV short-axes. A free-breathing, navigator-gated multi-echo sequence was used for short-axis T2 mapping. T1 mapping was performed using the modified Look-Locker inversion recovery (MOLLI) sequence. T2 maps were calculated from nine and T1 maps from eight echoes using a dedicated plug-in written for OsiriX software. Two experienced observers independently evaluated T2w-CMR as well as T1- and T2 mapping using the HeAT-Software applying a threshold method. Size of edema and prolongation of the native T1- or T2-time was measured using a cutoff >2SD of remote normal myocardium.

## Results

**Figure 1:**
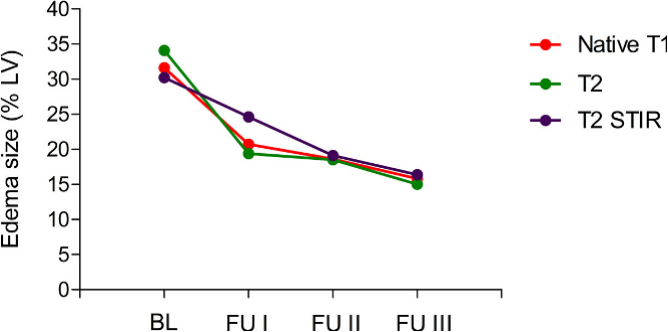
Figure 1

**Figure 2:**
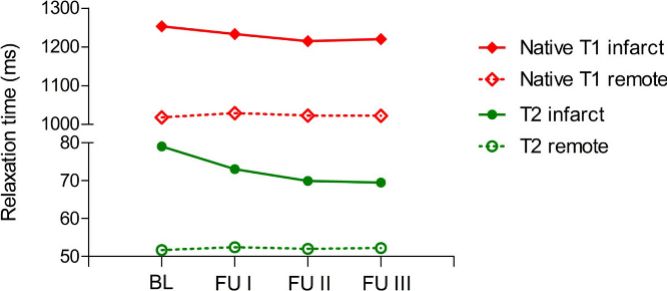
Figure 2

Edema size continuously decreased from BL with 32.8 %LV to 24.6 %LV at FU1, to 19.1 %LV at FU2 and to 16.4 %LV at FU3 using T2w-CMR (Figure 1). An almost identical decrease of edema size was observed using native T2- and T1-mapping (Figure 1). T2 times only decreased between BL from 79±5 ms to 73±2 ms at FU1 (*P*<0.05), but no further change was observed at later time points with 70±5 ms at FU2 and 70±6 ms at FU3. At all time points the T2 times of remote normal myocardium were about 50±2 ms and significantly lower compared to the edema zone (Figure 2). Also native T1 time within the edema was with 1253 ±103 ms significantly increased compared to remote normal myocardium with 1018 ± 43 ms and remained constantly high in the edema zone throughout all follow-ups (Figure 2).

## Conclusions

Edema size continuously decreased within the following months after acute myocardial infarction, but was still present after 6 months in all patients. Additionally, quantitative mapping show increased T2 and T1 values within the edema zone indicating prolonged presence of edema up to 6 months after infarction.

## Funding

This study is partially funded by the Deutsche Forschungsgemeinschaft.

